# 1,5-Bis­[1-(2-hy­droxy­phen­yl)ethyl­idene]carbonohydrazide dimethyl­formamide monosolvate

**DOI:** 10.1107/S1600536810037517

**Published:** 2010-09-30

**Authors:** Lingyun Du, Wenkang Zhang

**Affiliations:** a College of Chemistry and Chemical Engineering, Liaocheng University, Shandong 252059, People’s Republic of China

## Abstract

In the title compound, C_17_H_18_N_4_O_3_·C_3_H_7_NO, the main disubstituted urea and solvate mol­ecules are linked by pairs of N—H⋯O hydrogen bonds. In the main mol­ecules, the benzene rings form a dihedral angle of 15.59 (13)° a;nd two intra­molecular O—H⋯N hydrogen bonds influence the mol­ecular conformation. In the crystal structure, weak inter­molecular C—H⋯O inter­actions link the hydrogen-bonded pairs into chains along the *b* axis. The chains associate *via* C—H⋯π inter­actions.

## Related literature

For a related structure, see: Zukerman-Schpector *et al.* (2009 ▶). For the bioactivity of carbonohydrazide derivatives, see: Loncle *et al.* (2004 ▶); Li *et al.* (2004 ▶).
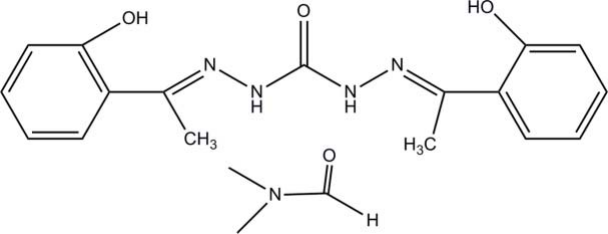


## Experimental

### 

#### Crystal data

C_17_H_18_N_4_O_3_·C_3_H_7_NO
*M* *_r_* = 399.45
Monoclinic, 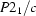

*a* = 16.6372 (15) Å
*b* = 7.5880 (9) Å
*c* = 16.2967 (14) Å
β = 94.472 (1)°
*V* = 2051.1 (4) Å^3^
*Z* = 4
Mo *K*α radiation
μ = 0.09 mm^−1^
*T* = 298 K
0.47 × 0.46 × 0.23 mm

#### Data collection

Bruker SMART APEX CCD area-detector diffractometer
Absorption correction: multi-scan (*SADABS*; Sheldrick, 1996 ▶) *T*_min_ = 0.958, *T*_max_ = 0.979
10277 measured reflections
3596 independent reflections
1712 reflections with *I* > 2σ(*I*)
*R*_int_ = 0.059

#### Refinement

*R*[*F* ^2^ > 2σ(*F* ^2^)] = 0.051
*wR*(*F* ^2^) = 0.165
*S* = 1.04
3596 reflections
263 parameters
H-atom parameters constrained
Δρ_max_ = 0.22 e Å^−3^
Δρ_min_ = −0.17 e Å^−3^

### 

Data collection: *SMART* (Bruker, 2007 ▶); cell refinement: *SAINT* (Bruker, 2007 ▶); data reduction: *SAINT*; program(s) used to solve structure: *SHELXS97* (Sheldrick, 2008 ▶); program(s) used to refine structure: *SHELXL97* (Sheldrick, 2008 ▶); molecular graphics: *SHELXTL* (Sheldrick, 2008 ▶); software used to prepare material for publication: *SHELXTL*.

## Supplementary Material

Crystal structure: contains datablocks I, global. DOI: 10.1107/S1600536810037517/cv2762sup1.cif

Structure factors: contains datablocks I. DOI: 10.1107/S1600536810037517/cv2762Isup2.hkl

Additional supplementary materials:  crystallographic information; 3D view; checkCIF report

## Figures and Tables

**Table 1:** Hydrogen-bond geometry (Å, °) *Cg* is the centroid of the C12–C17 ring.

*D*—H⋯*A*	*D*—H	H⋯*A*	*D*⋯*A*	*D*—H⋯*A*
N1—H1⋯O4	0.86	2.02	2.805 (3)	151
N3—H3⋯O4	0.86	2.09	2.858 (4)	148
O2—H2*A*⋯N2	0.82	1.83	2.548 (3)	145
O3—H3*A*⋯N4	0.82	1.83	2.546 (3)	145
C6—H6⋯O1^i^	0.93	2.57	3.241 (4)	129
C10—H10*A*⋯*Cg*^ii^	0.96	2.66	3.536 (4)	153

Symmetry codes: (i) 
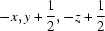
; (ii) 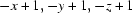.

**Fig. 1.**
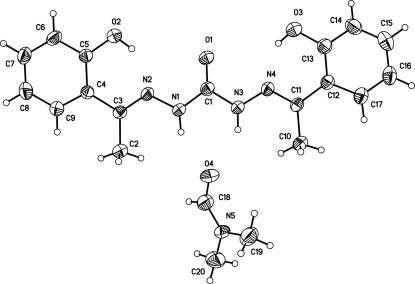
The content of asymmetric unit of the title compound showing the atomic numbering scheme and 30% probability displacement ellipsoids.

## supplementary crystallographic information

### Comment

Carbonohydrazide derivatives exhibit various bioactivities such as antibacteriale antifungal, anticonvulsant and anticancer activities (Loncle *et al.*, 2004; Li *et al.*, 2004). Herewith we present the crystal structure of the title compound (I), which is a new carbonohydrazide derivative.

Crystals of (I) comprise equal quantities of a disubstituted urea molecule (M) and a solvent N,*N*-dimethylformamide molecule (Fig. 1). The bond lengths and angles of the title compound are normal and correspond to those observed in *N*'',*N*'''-bis (1-(2-hydroxyphenyl)ethylidene)carbonohydrazide dimethyl sulfoxide solvate (Zukerman-Schpector *et al.*, 2009). The molecular conformation of M is influenced by two intramolecular O—H···N hydrogen bonds (Table 1). Two benzene  rings - C4-C9 and C12-C17, respectively - form a dihedral angle of 15.59 (13)°.

In the crystal structure, one M molecule and solvate molecule are paired *via* N—H···O hydrogen bonds (Table 1). Weak intermolecular C—H···O interactions (Table 1) link hydrogen-bonded pairs into chains along the *b* axis. The chains associate *via* C—H···π interactions (Table 1).

### Experimental

2-Hydroxylacetophenone (10.0 mmol) and carbohydrazide (5.0 mmol) were mixed in 50 ml flash After stirring 3 h at 373 K, the resulting mixture was cooled to room temperature, and recrystalized from DMF, and afforded the title compound as a crystalline solid. Elemental analysis: calculated for C_20_H_25_N_5_O_4_: C 60.14, H 6.31, N 17.53%; found: C 60.23, H 6.45, N 17.64%.

### Refinement

All H atoms were placed in geometrically idealized positions (N—H 0.86 and C—H = 0.93–0.96 Å, O—H= 0.82 Å) and treated as riding on their parent atoms, with *U*_iso_(H) = 1.2–1.5 *U*_eq_ of the parent atom.

### Crystal data

**Table:**

C_17_H_18_N_4_O_3_·C_3_H_7_NO	*F*(000) = 848
*M**_r_* = 399.45	*D*_x_ = 1.294 Mg m^−^^3^
Monoclinic, *P*2_1_/*c*	Mo *K*α radiation, λ = 0.71073 Å
*a* = 16.6372 (15) Å	Cell parameters from 1764 reflections
*b* = 7.5880 (9) Å	θ = 2.5–21.7°
*c* = 16.2967 (14) Å	µ = 0.09 mm^−^^1^
β = 94.472 (1)°	*T* = 298 K
*V* = 2051.1 (4) Å^3^	Block, colourless
*Z* = 4	0.47 × 0.46 × 0.23 mm

### Data collection

**Table:**

Bruker SMART APEX CCD area-detector diffractometer	3596 independent reflections
Radiation source: fine-focus sealed tube	1712 reflections with *I* > 2σ(*I*)
graphite	*R*_int_ = 0.059
phi and ω scans	θ_max_ = 25.0°, θ_min_ = 2.5°
Absorption correction: multi-scan (*SADABS*; Sheldrick, 1996)	*h* = −13→19
*T*_min_ = 0.958, *T*_max_ = 0.979	*k* = −9→9
10277 measured reflections	*l* = −19→19

### Refinement

**Table:**

Refinement on *F*^2^	Secondary atom site location: difference Fourier map
Least-squares matrix: full	Hydrogen site location: inferred from neighbouring sites
*R*[*F*^2^ > 2σ(*F*^2^)] = 0.051	H-atom parameters constrained
*wR*(*F*^2^) = 0.165	*w* = 1/[σ^2^(*F*_o_^2^) + (0.0542*P*)^2^ + 0.8303*P*] where *P* = (*F*_o_^2^ + 2*F*_c_^2^)/3
*S* = 1.04	(Δ/σ)_max_ < 0.001
3596 reflections	Δρ_max_ = 0.22 e Å^−^^3^
263 parameters	Δρ_min_ = −0.17 e Å^−^^3^
0 restraints	Extinction correction: *SHELXL*, Fc^*^=kFc[1+0.001xFc^2^λ^3^/sin(2θ)]^-1/4^
Primary atom site location: structure-invariant direct methods	Extinction coefficient: 0.0049 (10)

### Special details

**Table:**

Geometry. All e.s.d.'s (except the e.s.d. in the dihedral angle between two l.s. planes) are estimated using the full covariance matrix. The cell e.s.d.'s are taken into account individually in the estimation of e.s.d.'s in distances, angles and torsion angles; correlations between e.s.d.'s in cell parameters are only used when they are defined by crystal symmetry. An approximate (isotropic) treatment of cell e.s.d.'s is used for estimating e.s.d.'s involving l.s. planes.
Refinement. Refinement of *F*^2^ against ALL reflections. The weighted *R*-factor *wR* and goodness of fit *S* are based on *F*^2^, conventional *R*-factors *R* are based on *F*, with *F* set to zero for negative *F*^2^. The threshold expression of *F*^2^ > σ(*F*^2^) is used only for calculating *R*-factors(gt) *etc*. and is not relevant to the choice of reflections for refinement. *R*-factors based on *F*^2^ are statistically about twice as large as those based on *F*, and *R*- factors based on ALL data will be even larger.

### Fractional atomic coordinates and isotropic or equivalent isotropic displacement parameters (Å^2^)

**Table:**

	*x*	*y*	*z*	*U*_iso_*/*U*_eq_	
N1	0.15121 (15)	0.6195 (3)	0.48890 (15)	0.0541 (7)	
H1	0.1563	0.6254	0.5417	0.065*	
N2	0.08378 (15)	0.6805 (3)	0.44431 (15)	0.0491 (7)	
N3	0.27455 (15)	0.4912 (4)	0.49563 (15)	0.0555 (8)	
H3	0.2756	0.5015	0.5483	0.067*	
N4	0.33674 (15)	0.4171 (3)	0.45841 (15)	0.0510 (7)	
N5	0.23936 (17)	0.5436 (4)	0.78637 (16)	0.0602 (8)	
O1	0.20609 (13)	0.5357 (3)	0.37093 (14)	0.0717 (8)	
O2	0.02424 (14)	0.7754 (4)	0.30242 (13)	0.0772 (8)	
H2A	0.0592	0.7362	0.3357	0.116*	
O3	0.38946 (14)	0.3282 (4)	0.32185 (14)	0.0826 (8)	
H3A	0.3577	0.3729	0.3518	0.124*	
O4	0.22303 (16)	0.6047 (4)	0.65032 (15)	0.0825 (9)	
C1	0.20997 (19)	0.5488 (4)	0.4450 (2)	0.0511 (9)	
C2	0.0191 (2)	0.7253 (5)	0.57328 (18)	0.0606 (10)	
H2B	0.0483	0.6243	0.5950	0.091*	
H2C	−0.0361	0.7172	0.5863	0.091*	
H2D	0.0428	0.8305	0.5973	0.091*	
C3	0.02260 (19)	0.7313 (4)	0.48137 (18)	0.0445 (8)	
C4	−0.04633 (18)	0.7964 (4)	0.42688 (19)	0.0455 (8)	
C5	−0.04176 (19)	0.8178 (4)	0.3417 (2)	0.0516 (9)	
C6	−0.1065 (2)	0.8871 (5)	0.2935 (2)	0.0620 (10)	
H6	−0.1023	0.9033	0.2374	0.074*	
C7	−0.1762 (2)	0.9319 (5)	0.3270 (2)	0.0706 (11)	
H7	−0.2193	0.9779	0.2940	0.085*	
C8	−0.1824 (2)	0.9086 (5)	0.4098 (3)	0.0792 (12)	
H8	−0.2299	0.9381	0.4330	0.095*	
C9	−0.1183 (2)	0.8417 (5)	0.4584 (2)	0.0634 (10)	
H9	−0.1235	0.8264	0.5143	0.076*	
C10	0.40492 (19)	0.3582 (5)	0.59426 (19)	0.0617 (10)	
H10A	0.4233	0.4721	0.6132	0.093*	
H10B	0.4426	0.2699	0.6148	0.093*	
H10C	0.3531	0.3346	0.6139	0.093*	
C11	0.39830 (18)	0.3552 (4)	0.50206 (19)	0.0470 (8)	
C12	0.46219 (18)	0.2798 (4)	0.4551 (2)	0.0485 (8)	
C13	0.4547 (2)	0.2668 (5)	0.3684 (2)	0.0588 (9)	
C14	0.5149 (2)	0.1875 (5)	0.3275 (2)	0.0756 (12)	
H14	0.5082	0.1747	0.2706	0.091*	
C15	0.5839 (2)	0.1277 (5)	0.3690 (3)	0.0799 (12)	
H15	0.6244	0.0772	0.3404	0.096*	
C16	0.5933 (2)	0.1423 (5)	0.4528 (3)	0.0738 (11)	
H16	0.6404	0.1021	0.4813	0.089*	
C17	0.5336 (2)	0.2159 (4)	0.4947 (2)	0.0610 (10)	
H17	0.5410	0.2237	0.5517	0.073*	
C18	0.1982 (2)	0.5477 (5)	0.7144 (2)	0.0700 (11)	
H18	0.1458	0.5041	0.7116	0.084*	
C19	0.3198 (2)	0.6151 (6)	0.7959 (2)	0.0948 (14)	
H19A	0.3380	0.6399	0.7427	0.142*	
H19B	0.3553	0.5311	0.8239	0.142*	
H19C	0.3195	0.7218	0.8275	0.142*	
C20	0.2055 (2)	0.4702 (6)	0.8576 (2)	0.0931 (14)	
H20A	0.1503	0.4381	0.8436	0.140*	
H20B	0.2080	0.5561	0.9010	0.140*	
H20C	0.2356	0.3676	0.8757	0.140*	

### Atomic displacement parameters (Å^2^)

**Table:**

	*U*^11^	*U*^22^	*U*^33^	*U*^12^	*U*^13^	*U*^23^
N1	0.0494 (16)	0.072 (2)	0.0391 (15)	0.0127 (15)	−0.0055 (13)	−0.0015 (14)
N2	0.0449 (16)	0.0560 (18)	0.0448 (16)	0.0048 (14)	−0.0071 (14)	−0.0012 (13)
N3	0.0466 (16)	0.077 (2)	0.0421 (15)	0.0101 (15)	−0.0048 (13)	−0.0037 (15)
N4	0.0432 (15)	0.0593 (18)	0.0493 (17)	0.0022 (14)	−0.0032 (13)	−0.0033 (14)
N5	0.0661 (19)	0.073 (2)	0.0406 (17)	0.0032 (17)	0.0014 (15)	0.0090 (15)
O1	0.0632 (15)	0.106 (2)	0.0436 (15)	0.0167 (14)	−0.0101 (12)	−0.0117 (14)
O2	0.0669 (16)	0.117 (2)	0.0473 (14)	0.0275 (15)	0.0014 (13)	0.0008 (14)
O3	0.0693 (17)	0.125 (2)	0.0517 (15)	0.0146 (16)	−0.0044 (13)	−0.0063 (15)
O4	0.099 (2)	0.107 (2)	0.0418 (15)	0.0096 (17)	0.0026 (14)	0.0109 (15)
C1	0.0455 (19)	0.058 (2)	0.048 (2)	−0.0022 (17)	−0.0055 (17)	−0.0060 (18)
C2	0.064 (2)	0.068 (2)	0.049 (2)	0.0049 (19)	−0.0020 (17)	0.0008 (18)
C3	0.049 (2)	0.042 (2)	0.0421 (19)	−0.0037 (16)	−0.0026 (16)	−0.0045 (15)
C4	0.0480 (19)	0.0426 (19)	0.045 (2)	0.0011 (16)	−0.0012 (16)	−0.0055 (16)
C5	0.051 (2)	0.058 (2)	0.044 (2)	0.0062 (17)	−0.0033 (17)	−0.0065 (17)
C6	0.067 (2)	0.068 (3)	0.048 (2)	0.007 (2)	−0.0143 (19)	−0.0001 (18)
C7	0.058 (2)	0.081 (3)	0.070 (3)	0.019 (2)	−0.015 (2)	−0.005 (2)
C8	0.053 (2)	0.103 (3)	0.080 (3)	0.022 (2)	0.004 (2)	−0.006 (3)
C9	0.057 (2)	0.078 (3)	0.055 (2)	0.011 (2)	0.0026 (19)	−0.001 (2)
C10	0.061 (2)	0.070 (2)	0.053 (2)	0.0082 (19)	−0.0060 (17)	0.0022 (19)
C11	0.0440 (19)	0.047 (2)	0.049 (2)	−0.0037 (16)	−0.0047 (16)	−0.0020 (16)
C12	0.045 (2)	0.046 (2)	0.054 (2)	−0.0063 (16)	−0.0020 (17)	0.0011 (17)
C13	0.053 (2)	0.065 (2)	0.057 (2)	−0.0051 (19)	−0.0014 (19)	−0.0055 (19)
C14	0.070 (3)	0.093 (3)	0.066 (3)	−0.004 (2)	0.017 (2)	−0.012 (2)
C15	0.068 (3)	0.074 (3)	0.101 (4)	0.006 (2)	0.028 (3)	−0.004 (3)
C16	0.055 (2)	0.072 (3)	0.094 (3)	0.012 (2)	0.004 (2)	0.011 (2)
C17	0.055 (2)	0.058 (2)	0.070 (2)	0.0031 (19)	−0.001 (2)	0.0056 (19)
C18	0.070 (3)	0.075 (3)	0.064 (3)	0.000 (2)	−0.001 (2)	−0.001 (2)
C19	0.072 (3)	0.132 (4)	0.078 (3)	−0.003 (3)	−0.009 (2)	0.010 (3)
C20	0.116 (3)	0.100 (3)	0.066 (3)	0.013 (3)	0.024 (3)	0.025 (2)

### Geometric parameters (Å, °)

**Table:**

N1—C1	1.365 (4)	C7—C8	1.372 (5)
N1—N2	1.369 (3)	C7—H7	0.9300
N1—H1	0.8600	C8—C9	1.375 (5)
N2—C3	1.283 (4)	C8—H8	0.9302
N3—N4	1.361 (3)	C9—H9	0.9300
N3—C1	1.374 (4)	C10—C11	1.498 (4)
N3—H3	0.8600	C10—H10A	0.9600
N4—C11	1.289 (4)	C10—H10B	0.9600
N5—C18	1.311 (4)	C10—H10C	0.9600
N5—C19	1.440 (4)	C11—C12	1.473 (4)
N5—C20	1.441 (4)	C12—C17	1.394 (4)
O1—C1	1.208 (3)	C12—C13	1.411 (4)
O2—C5	1.352 (3)	C13—C14	1.384 (5)
O2—H2A	0.8200	C14—C15	1.364 (5)
O3—C13	1.358 (4)	C14—H14	0.9300
O3—H3A	0.8200	C15—C16	1.367 (5)
O4—C18	1.232 (4)	C15—H15	0.9299
C2—C3	1.504 (4)	C16—C17	1.368 (5)
C2—H2B	0.9600	C16—H16	0.9300
C2—H2C	0.9600	C17—H17	0.9300
C2—H2D	0.9600	C18—H18	0.9300
C3—C4	1.480 (4)	C19—H19A	0.9600
C4—C9	1.382 (4)	C19—H19B	0.9600
C4—C5	1.405 (4)	C19—H19C	0.9600
C5—C6	1.387 (4)	C20—H20A	0.9600
C6—C7	1.363 (5)	C20—H20B	0.9600
C6—H6	0.9300	C20—H20C	0.9600
			
C1—N1—N2	116.4 (3)	C11—C10—H10A	109.5
C1—N1—H1	121.8	C11—C10—H10B	109.5
N2—N1—H1	121.8	H10A—C10—H10B	109.5
C3—N2—N1	120.0 (3)	C11—C10—H10C	109.5
N4—N3—C1	116.7 (3)	H10A—C10—H10C	109.5
N4—N3—H3	121.6	H10B—C10—H10C	109.5
C1—N3—H3	121.6	N4—C11—C12	115.4 (3)
C11—N4—N3	120.2 (3)	N4—C11—C10	122.7 (3)
C18—N5—C19	120.2 (3)	C12—C11—C10	121.8 (3)
C18—N5—C20	121.3 (3)	C17—C12—C13	116.4 (3)
C19—N5—C20	118.5 (3)	C17—C12—C11	121.2 (3)
C5—O2—H2A	109.5	C13—C12—C11	122.4 (3)
C13—O3—H3A	109.5	O3—C13—C14	117.2 (3)
O1—C1—N1	124.8 (3)	O3—C13—C12	122.7 (3)
O1—C1—N3	123.5 (3)	C14—C13—C12	120.1 (3)
N1—C1—N3	111.6 (3)	C15—C14—C13	121.2 (4)
C3—C2—H2B	109.5	C15—C14—H14	119.4
C3—C2—H2C	109.5	C13—C14—H14	119.4
H2B—C2—H2C	109.5	C14—C15—C16	119.7 (4)
C3—C2—H2D	109.5	C14—C15—CG1	59.7 (2)
H2B—C2—H2D	109.5	C14—C15—H15	120.2
H2C—C2—H2D	109.5	C16—C15—H15	120.2
N2—C3—C4	115.1 (3)	C15—C16—C17	120.0 (4)
N2—C3—C2	123.6 (3)	C15—C16—H16	120.0
C4—C3—C2	121.3 (3)	C17—C16—H16	120.0
C9—C4—C5	116.9 (3)	C16—C17—C12	122.5 (4)
C9—C4—C3	120.9 (3)	C16—C17—H17	118.8
C5—C4—C3	122.2 (3)	C12—C17—H17	118.8
O2—C5—C6	116.4 (3)	O4—C18—N5	125.4 (4)
O2—C5—C4	123.3 (3)	O4—C18—H18	117.3
C6—C5—C4	120.3 (3)	N5—C18—H18	117.3
C7—C6—C5	121.0 (3)	N5—C19—H19A	109.5
C7—C6—H6	119.5	N5—C19—H19B	109.5
C5—C6—H6	119.5	H19A—C19—H19B	109.5
C6—C7—C8	119.6 (3)	N5—C19—H19C	109.5
C6—C7—H7	120.2	H19A—C19—H19C	109.5
C8—C7—H7	120.2	H19B—C19—H19C	109.5
C7—C8—C9	120.0 (4)	N5—C20—H20A	109.5
C7—C8—H8	120.0	N5—C20—H20B	109.5
C9—C8—H8	120.0	H20A—C20—H20B	109.5
C8—C9—C4	122.2 (3)	N5—C20—H20C	109.5
C8—C9—H9	118.9	H20A—C20—H20C	109.5
C4—C9—H9	118.9	H20B—C20—H20C	109.5

### Hydrogen-bond geometry (Å, °)

**Table:**

Cg is the centroid of the C12–C17 ring.

**Table:**

*D*—H···*A*	*D*—H	H···*A*	*D*···*A*	*D*—H···*A*
N1—H1···O4	0.86	2.02	2.805 (3)	151
N3—H3···O4	0.86	2.09	2.858 (4)	148
O2—H2A···N2	0.82	1.83	2.548 (3)	145
O3—H3A···N4	0.82	1.83	2.546 (3)	145
C6—H6···O1^i^	0.93	2.57	3.241 (4)	129
C10—H10A···Cg^ii^	0.96	2.66	3.536 (4)	153

Symmetry codes: (i) −*x*, *y*+1/2, −*z*+1/2; (ii) −*x*+1, −*y*+1, −*z*+1.
